# Os odontoideum associated with a retro-odontoid cyst treated with posterior C1–C3 fixation: A case report and literature review

**DOI:** 10.3389/fsurg.2022.1006167

**Published:** 2023-01-06

**Authors:** Bei-Xi Bao, Hui Yan, Jia-Guang Tang

**Affiliations:** Department of Orthopedic Surgery, Beijing Tongren Hospital, Capital Medical University, Beijing, China

**Keywords:** os odontoideum, retro-odontoid cyst, posterior c1-C3 fixation, atlantoaxial stability, gradual resorption of cyst

## Abstract

**Background:**

Os odontoideum is a rare abnormality of the upper cervical spine, and os odontoideum associated with a retro-odontoid cyst has been described as a marker of local instability.

**Case description:**

This paper reports a case of a 52-year-old female patient who was diagnosed with os odontoideum associated with a retro-odontoid cyst. The patient underwent posterior C1–C3 fixation without surgical removal of the cyst. Magnetic resonance imaging (MRI) two days later revealed that the retro-odontoid cyst was still present and that there were no significant changes to it when compared with the preoperative MRI.

**Conclusion:**

Retro-odontoid cysts associated with unstable os odontoideum can lead to symptomatic spinal cord compression. Posterior C1–C3 fixation can restore atlantoaxial stability by allowing the gradual resorption of the cyst and ensuring spinal cord decompression. Fixation can also avoid the surgical risk associated with a high-riding vertebral artery.

## Introduction

Os odontoideum is a rare malformation of the upper cervical spine, and its combination with a retro-odontoid cyst has been described as a marker of local instability. Different terms are associated with retro-odontoid structures involving spinal cord compression, including mass, cyst, pseudotumor, granuloma, and pannus. It is also thought that the atlantoaxial instability related to os odontoideum may prompt the appearance of a retro-odontoid cyst. The nonspecific symptomatic indications of the two together are erratic and vary from cervical pain to neurological disorders (1, 2).

This paper reports a case of os odontoideum associated with a retro-odontoid cyst that was treated with posterior C1–C3 fixation without surgical removal of the cyst. Using magnetic resonance imaging (MRI) that was performed immediately and three months after the fixation operation, it was found, for the first time, that the retro-odontoid cyst did not disappear immediately but was gradually resorbed.

## Case report

**Figure 1:**
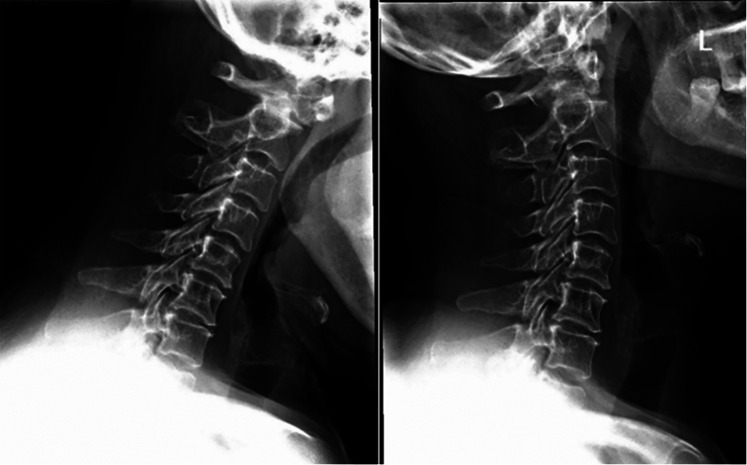
Preoperative x-ray revealing atlantoaxial instability.

**Figure 2:**
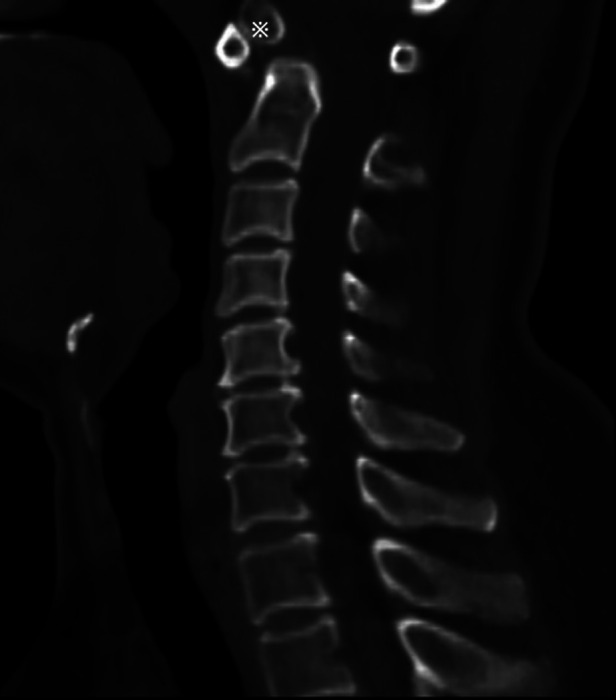
Sagittal CT view showing os odontoideum (※).

A 52-year-old female patient had displayed numbness (paresthesia) and weakness in her limbs for 3 months. No hemodynamic or respiratory complications were observed during her initial care, and a neurological examination indicated a AIS was D (American Spinal Injury Association Impairment Scale) (3) and no pyramidal syndrome. Cervical flexion and extension radiographs suggested atlantoaxial instability (Figure 1), and a cervical computed tomography (CT) scan demonstrated the existence of a bony continuity that had not been displaced within the odontoid process (Figure 2). These findings, along with the existence of corticalized edges, led to a diagnosis of os odontoideum.

**Figure 3:**
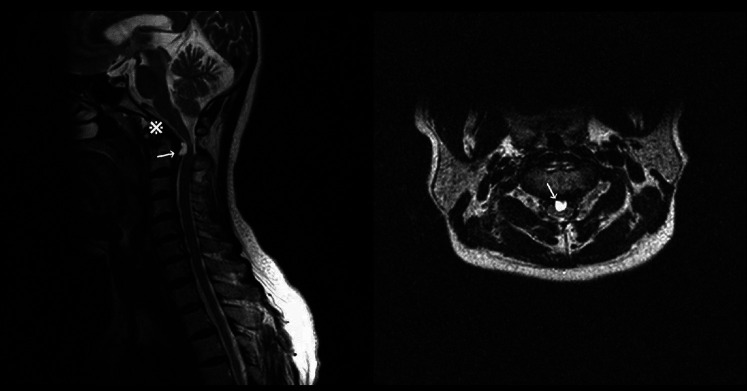
Sagittal and axial preoperative magnetic resonance imaging showing os odontoideum (※) and a compressive retro-odontoid cyst (arrows) with a hyperintense medullary signal.

**Figure 4:**
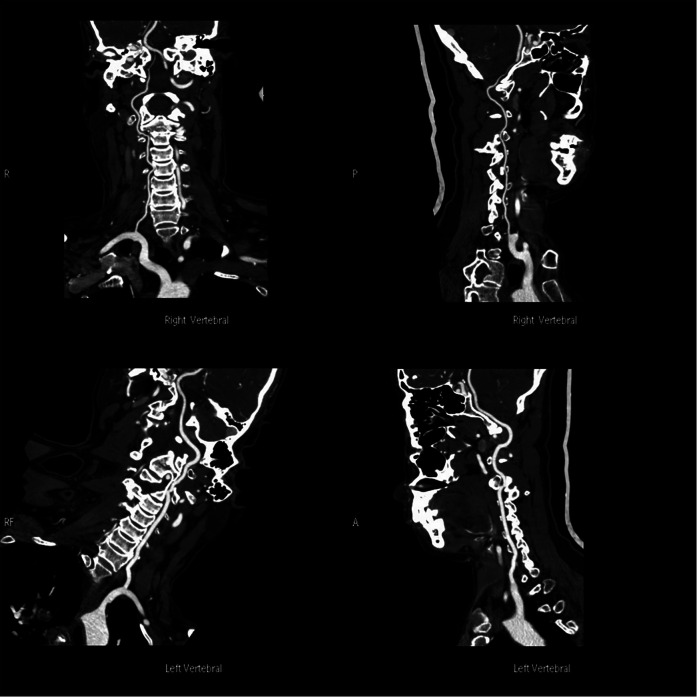
Preoperative carotid CTA indicated the presence of a high left carotid artery span in the patient.

To evaluate the existence of any recent neurological disorders, a cervical MRI was performed, which led to the discovery of a retro-odontoid cyst with T2 hyperintensity and T1 hypointensity. The cyst was exerting pressure on the spinal cord, and the intramedullary T2 hyperintensity signified a spinal cord disorder (Figure 3). Figure 4 indicates the presence of a high left carotid artery span in the patient.

**Figure 5:**
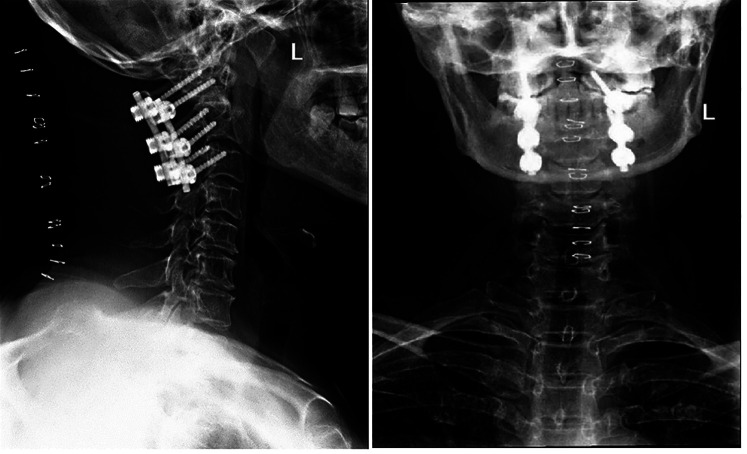
Postoperative lateral x-ray of cervical spine.

**Figure 6:**
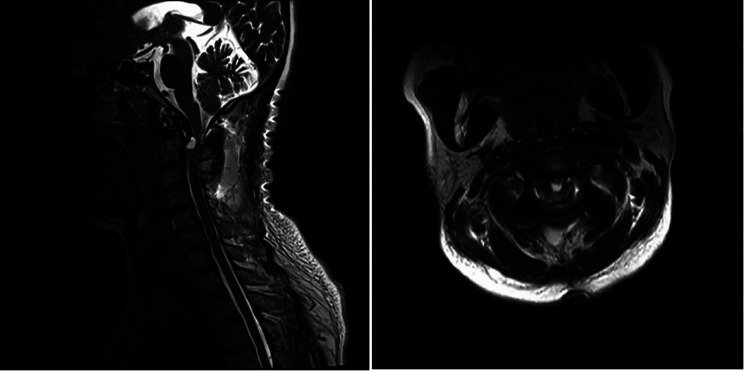
Sagittal and axial immediate postoperative magnetic resonance imaging showing the continued presence of the retro-odontoid cyst.

**Figure 7:**
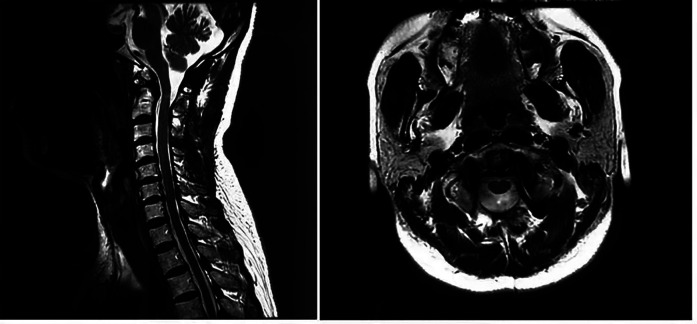
Sagittal and axial 3-month postoperative magnetic resonance imaging showing the complete disappearance of the retro-odontoid cyst.

It was decided to perform a C1–C3 fixation (Figure 5), and, two days after the operation, the MRI results showed that the retro-odontoid cyst was still present and had not changed significantly compared with the preoperative MRI results (Figure 6). However, the patient's clinical progress was rapid and favorable, with the paresthesia regressing and motor recovery reaching grade 4 out of 5 by MRC (Medical Research Council) Scale for Muscle Strength (4). She was discharged one week after the surgery with no scarring and no longer presenting paresthesia in the right upper limb, and her ASIA was E. At 3 months, a complete neurological recovery was confirmed. Some cervical pain was reported, 2/10 on the VAS (Visual Analog Scale) (5), without it being debilitating. The follow-up MRI also showed the complete disappearance of the retro-odontoid cyst and no signs of persistent spinal cord involvement (Figure 7).

## Discussion

### Os odontoideum and a retro-odontoid cyst

Os odontoideum was first described by Giacomini in 1886. This abnormality is an anatomical variation of the C2 odontoid process and defined as a small bone of varying size with a smooth margin of the surrounding cortex, separated from the axis.

The etiology of os odontoideum remains controversial but is focused on congenital and acquired causes. With respect to the former, the disorder is often associated with congenital malformations such as Klippel–Feil syndrome, trisomy 21 syndrome, Morquio syndrome, or neurofibromatosis. In terms of the latter, an insufficient arterial blood supply and unrecognized or repeated minor trauma, resulting in transverse ligament contracture, have been found to result in secondary odontoid base contraction (6, 7).

A synovial cyst is a cystic mass lined with pseudo-lamellar columnar cells and containing clear or yellow fluid. Although synovial cysts often occur in the lumbar spine, they rarely occur in the inferior cervical axis and rarely involve the odontoid process. The cause of posterior odontoid cysts is unknown, but previous studies have suggested that these cysts are associated with growth of synovial rests, pluripotent stromal cell proliferation, atlantoaxial instability, and trauma (8). The association of os odontoideum with a posterior odontoid cyst has been described as a marker of local instability, and atlantoaxial instability associated with the odontoid bone may also suggest a retro-odontoid cyst.

Computed tomography and MRI are of great significance in the diagnosis of this type of cyst, which produces a low T1 signal and a high T2 signal. Cysts may also appear black on T2-weighted MRI, possibly due to bleeding and blood products. Peripheral angiographic enhancement is also common, which may be linked to the adjacent epidural plexus. However, cervical synovial cysts cannot be accurately distinguished from other lesions by MRI features, and in many cases, accurate diagnosis can only be made after surgery (1, 2, 8–32).

### Treatment

Hypoplasia of the odontoid associated with an independent oval ossicle, with smooth margins widely separated from the C2 vertebra and well above the superior facets of the axis, is termed os odontoideum. It is a rare condition with a controversial pathogenesis and poorly understood natural history. The neurological manifestations of os odontoideum arise from bulbospinal compression both at rest and during motion due to craniovertebral junction (CVJ) instability. Consequently, the surgical management of os odontoideum should aim at achieving both neural decompression and stabilization of the CVJ (33).

With regard to anterior decompressive procedures, in particular, it has been widely reported that alternative transoral decompressive strategies have been improved with the introduction of endoscopy, neuronavigation, exoscopy, and intraoperative neuroradiological assessment such as the O-arm CT scan. All these approaches contribute to reducing the invasiveness of decompressive procedures and increasing their effectiveness (34–37). In addition, although the transnasal approach seems a more promising minimally invasive alternative to the transoral approach, it can lead to some adverse effects that deserve consideration, including cerebrospinal fluid leakage, infections, and velopalatal dysfunction (38–41). For these reasons, posterior atlantoaxial fixation seems to be a more promising and less invasive form of treatment, but it should only be used for bioptical procedures. In 2004, Goel reported two patients with Atlantoaxial instability and retroodontoid mass. Following atlantoaxial fixation, both patients showed remarkable and sustained neurological improvement. These cases provide further evidence that retroodontoid ligamentous hypertrophic mass lesion could be secondary to instability of the atlantoaxial region (42). Goel (43) also reported an experience with 190 cases of os odontoideum over 20 years. He thought the segmental atlantoaxial fixation is a reliable form of surgical treatment. Bone decompression is not necessary. Inclusion of occipital bone and subaxial vertebrae in the fixation construct is not necessary.

In 2021, Goel (44) searched 63 patients with retro-odontoid pseudotumor, pannus, and/or cyst (RPC) treated by atlantoaxial fixation from January 2000 to March 2020. After atlantoaxial stabilization, the RPC spontaneously regressed or disappeared. Direct resection of the RPC was neither performed nor was necessary in any case. In our case, due to the presence of a high-riding vertebral artery in the patient's preoperative CT examination and to avoid injury to it during screw placement in the C2 vertebra, relatively short screws were used, and posterior C1–C3 fixation was performed to ensure the stability of the fixation. The procedure reconfirmed that retro-odontoid cysts can be treated by posterior spinal fixation and that there is no need to remove the cyst directly by surgery.

In the case reported here, the immediate postoperative MRI showed that the retro-odontoid cyst was still present and had not changed significantly from that of the preoperative MRI findings. However, at the 3-month postoperative follow-up, the MRI confirmed the complete disappearance of the retro-odontoid cyst and the decompression of the spinal cord. This demonstrated, for the first time, that the retro-odontoid cyst did not disappear immediately after surgery but was gradually resorbed.

## Conclusion

Retro-odontoid cysts associated with unstable os odontoideum can lead to symptomatic spinal cord compression, but posterior C1–C3 fixation can restore atlantoaxial stability. This approach will allow for the gradual resorption of the cyst postoperatively and ensure spinal cord decompression, and it can also avoid the inherent risks of an operation to remove a cyst when the vertebral artery is high-riding.

## Data Availability

The original contributions presented in the study are included in the article/Supplementary Material, further inquiries can be directed to the corresponding author/s.
